# Associations between exposure to organophosphate esters and overactive bladder in U.S. adults: a cross-sectional study

**DOI:** 10.3389/fpubh.2023.1186848

**Published:** 2023-11-09

**Authors:** Weilong Lin, Haoxu Wang, Zesong Wu, Wei Zhang, Ming-En Lin

**Affiliations:** ^1^The First Affiliated Hospital of Shantou University Medical College, Medical College of Shantou University, Shantou, Guangdong, China; ^2^Clinical Medicine Science, Shantou University Medical College, Shantou, Guangdong, China; ^3^The First Affiliated Hospital of Shantou University Medical College Hao Jiang Hospital, Shantou, Guangdong, China

**Keywords:** organophosphate esters (OPEs), overactive bladder (OAB), NHANES, nocturia, urge urinary incontinence

## Abstract

**Background:**

The relationship between exposure to organophosphate esters (OPEs) and the risk of developing overactive bladder (OAB) is uncertain. The purpose of this study is to examine the potential link between urinary metabolites of organophosphate esters and OAB.

**Method:**

Data from the National Health and Nutrition Examination Survey (NHANES) database of the 2011–2016 cycles were utilized. Four urinary metabolites of organophosphate esters: diphenyl phosphate (DPHP), bis (1,3-dichloro-2-propyl) phosphate (BDCPP), bis (2-chloroethyl) phosphate (BCEP), and dibutyl phosphate (DBUP) were included in the study. Multivariate logistic regression and restricted cubic spline (RCS) were used to evaluate the relationship between urinary OPEs metabolites and OAB. Interaction analysis was conducted on subgroups to confirm the findings.

**Results:**

A total of 3,443 United States (US) adults aged 20 years or older were included in the study, of whom 597 participants were considered to have OAB. After adjusting for potential confounding factors, we found a positive association between DPHP and the risk of overactive bladder. The risk of overactive bladder increased with increasing DPHP concentrations compared with quartile 1 (quartile 2, OR = 1.19, 95% CI, 0.82–1.73, *P* = 0.34; quartile 3, OR = 1.67, 95% CI, 1.10–2.53, *P* = 0.02; Q4, OR = 1.75, 95% CI, 1.26–2.43, *P* = 0.002). However, after dividing the participants by gender, only the female group retained consistent results. Additionally, restricted cubic spline analysis revealed a nonlinear dose-response correlation between DPHP and OAB in female participants. In the subgroup analysis based on age, race, body mass index (BMI), recreational activity, smoking status, drinking status, hypertension, diabetes, and stroke, the interaction analysis revealed that the findings were uniform.

**Conclusion:**

Our findings indicate that exposure to DPHP could elevate the risk of OAB in US adult females. Further experimental studies are needed to explore the underlying mechanism in the future.

## 1. Introduction

As a substitute for customary brominated flame retardants (BFRs), organophosphate esters (OPEs) have been extensively utilized in an array of products, comprising building materials, electronics, and furniture (1, 2). Correspondingly, OPEs could also be implemented as plasticizers in different consumer products, like cosmetics and children's commodities (3, 4). With the gradual elimination of customary brominated flame retardants, the worldwide yield and utilization of organophosphates have grown enormously over the previous 15 years (5). The human body may be exposed to OPEs through indoor dust, drinking water, dietary intake, etc (6–8), which leads to constant human exposure to OPEs. And high concentration, long-term exposure to OPEs will lead to several adverse effects. Presently, animal experimental studies have shown that tris(1, 3dichloro-2-propyl) phosphate (TDCPP) can diminish serum thyroxine levels in chicken embryos (9). And exposure to higher concentrations of tris(1, 3-dichloro-2-isopropyl) phosphate (TDCIPP) is prone to hyperthyroidism in domestic cats (10). Tri-ortho-cresyl phosphate (TOCP) can destroy the spermatogenic epithelium of mouse testicles and reduce sperm density (11). It is noteworthy that some halogenated OPEs are carcinogenic to animals (12). Furthermore, studies indicate that OPEs increase the risk of cardiovascular disease, metabolic syndrome, urinary incontinence, and non-alcoholic fatty liver disease in adults (13–16). Meanwhile, in adolescents, it can also cause disorders of glucose metabolism, induce pre-diabetes, and lead to hypertension (17, 18).

Overactive bladder (OAB) was defined by the International Continence Society (ICS) in 2002 as a storage symptom syndrome characterized by “urgency, with or without urgency urinary incontinence (UUI), usually with increased daytime frequency and nocturia” (19). An epidemiological survey revealed that the overall prevalence of OAB in four European countries and Canada was 11.8%, with no significant difference between males and females (20). Surveys conducted in the United States indicate that the prevalence of OAB in the American population is 16.0% for males and 16.9% for females and that this increases with age (21). As a globally prevalent chronic disease, OAB significantly impacts patients' physical health and quality of life (22). Moreover, the healthcare costs of patients with OAB in the US population were >2.5 times higher than those of similar patients without OAB (23). However, the pathophysiology of OAB is currently unclear (24). Certain studies have indicated that OAB may be associated with risk factors such as obesity, smoking, alcohol consumption, reduced physical activity, diabetes, depression, and lower socioeconomic status (25–27). Previous research has demonstrated that exposure to environmental pollutants, including di-(2-ethylhexyl) phthalate, may elevate the probability of OAB (28). Nevertheless, no studies have concentrated on the correlation between exposure to OPEs and OAB or investigated the dose-response relationship between human metabolites of OPEs and OAB. Accordingly, we have utilized data from the National Health and Nutrition Examination Surveys (NHANES) from the 2011 to 2016 cycles to investigate the relationships.

## 2. Methods

### 2.1. Study population

The National Health and Nutrition Examination Survey (NHANES) is a continuous stratified multi-stage sampling program designed to evaluate the health and nutritional condition of adults and children in the United States, which involves a variety of health and nutrition measures. NHANES surveys a nationally representative sample of about 5,000 people each year, consisting mainly of interviews and physical examinations. The interview section includes demographic, socioeconomic, dietary, and health-related questions. The physical examination part includes physiological measurements, laboratory tests, etc. Written informed consent was obtained from each participant. The NHANES research project was reviewed and approved by the National Center for Health Statistics Research Board.

Our analysis involved studying data from NHANES over 6 years (2011–2012, 2013–2014, 2015–2016) and included 29,902 participants. After excluding participants with missing data on OPE urine metabolites or urine creatinine (*n* = 22,761), incomplete data on OAB-related questionnaires (*n* = 2,447) and covariates (*n* = 654). Meanwhile, participants who were pregnant during the interview (*n* = 33), participants who reported a weak/failing kidney (*n* = 150), and participants with a history of cancer or malignancy (*n* = 414) were also excluded. Finally, a total of 3,443 participants were included in the study.

### 2.2. Urine OPEs metabolites assessment

In NHANES, a random selection was made of one-third of participants older than 6 years for the measurement of organophosphate metabolites. Then 0.2 mL of the subject's urine was used for enzymatic hydrolysis of urinary conjugates of the target analytes, automated off-line solid phase extraction, reversed phase high-performance liquid chromatography separation, and isotope dilution-electrospray ionization tandem mass spectrometry detection (29). We included four major OPE urine metabolites in this study (15): Diphenyl phosphate(DPHP), Bis(1,3-dichloro-2-propyl)phosphate(BDCPP), Bis(2-chloroethyl) phosphate(BCEP), and Dibutyl phosphate(DBUP). According to the NHANES Analytic Guidelines, if the detection results of urine metabolites were below the limit of detection, the values were imputed by the square root of two. Meantime, to account for variation in urine sample volume, the OPEs were adjusted by urine creatinine using the values of OPEs/creatinine (μg/g) as the analytical variables.

### 2.3. Assessment of OAB

According to the definition of OAB, the presence of OAB should be considered when the patient has urge urinary incontinence and nocturia. We used the following three questions from the Kidney Conditions-Urology questionnaire in NHANES: (1) During the past 12 months, have you leaked or lost control of even a small amount of urine with an urge or pressure to urinate and you couldn't get to the toilet fast enough? (2) How frequently does this occur? (3) During the past 30 days, how many times per night did you most typically get up to urinate, from the time you went to bed at night until the time you got up in the morning? We leveraged the table of the Criteria for Conversion of Symptom Frequencies Recorded in NHANES and Overactive Bladder Symptom Score (OABSS) Scores, as utilized by Shenhao Zhu et al. (30). To enhance the diagnosis of OAB, we further quantified overactive bladder symptoms by specific scoring refer to Table 1 for specific scoring criteria. Finally, each participant's overall OABSS score was obtained by adding the nocturia score and the urge urinary incontinence score. Individuals with a total score ≥3 were considered to have a diagnosis of overactive bladder disorder (30).

**Table 1:** Criteria for conversion of symptom frequencies recorded in NHANES and OABSS scores.

**According to NHANES score**	**According to OABSS score**
**Urge urinary incontinence frequency**	**Urge urinary incontinence score**
Never	0
Less than once a month	1
A few times a month	1
A few times a week	2
Every day or night	3
**Nocturia frequency Nocturia score**	**Nocturia frequency Nocturia score**
0	0
1	1
2	2
3	3
4	3
5 or more	3

NHANES, National Health and Nutrition Examination Survey; OABSS, Overactive Bladder Symptom Score.

### 2.4. Covariates

We used a Directed Acyclic Graph (DAG) (www.dagitty.net/dags.html) to show the hypothesized relations between OPEs, confounders, and overactive bladder outcomes (Supplementary Figure 1). According to the DAG, participants' sex, age, race, body mass index (BMI), education level, poverty income ratio (PIR), recreational activity, smoking, and drinking were included as confounding factors. We also carefully considered alternative DAG that included more variables, such as variables related to OAB or OPEs. Marital status, hypertension, and diabetes were also included as relevant variables.

### 2.5. Statistical analysis

To reduce the effects of the complex multistage sampling design of NHANES, we used appropriate sample weights to improve data precision, according to NHANES guidelines. Urinary creatinine-corrected metabolite concentrations of OPEs were natural-log transformed (ln-transformed) due to skewed distribution and categorized into four quartiles (Q1, Q2, Q3, and Q4). Demographic characteristics were expressed in weighted mean ± standard error (SE) for continuous variables and weighted percentage (%) for categorical variables by OAB status. Weighted *t*-tests for continuous variables and weighted chi-squared tests for categorical variables were used to assess the baseline characteristics of the participants by OAB status. Multivariable logistic regression analyses were utilized to explore the relationships between OPEs and OAB status. We constructed three logistic regression models: model 1 adjusted for no variables; model 2 adjusted for age, sex, race, education level, PIR, and BMI; model 3 further adjusted for recreational activity, smoking status, drinking status, hypertension, diabetes as well as stroke. In addition, in Model 3, we included OPEs urine concentration as a continuous variable and used a restricted cubic spline (RCS) to reveal the dose-response relationship between OPEs concentration and OAB risk. We then used subgroup analyses stratified by age, race, BMI, recreational activity, smoking status, drinking status, hypertension, diabetes, and stroke, and performed interaction analyses to examine whether there was a differential association between subgroups. All statistical analyses in this study were organized and analyzed using R software (R 4.2.3). Two-sided P < 0.05 was considered statistically significant.

## 3. Result

**Figure 1:**
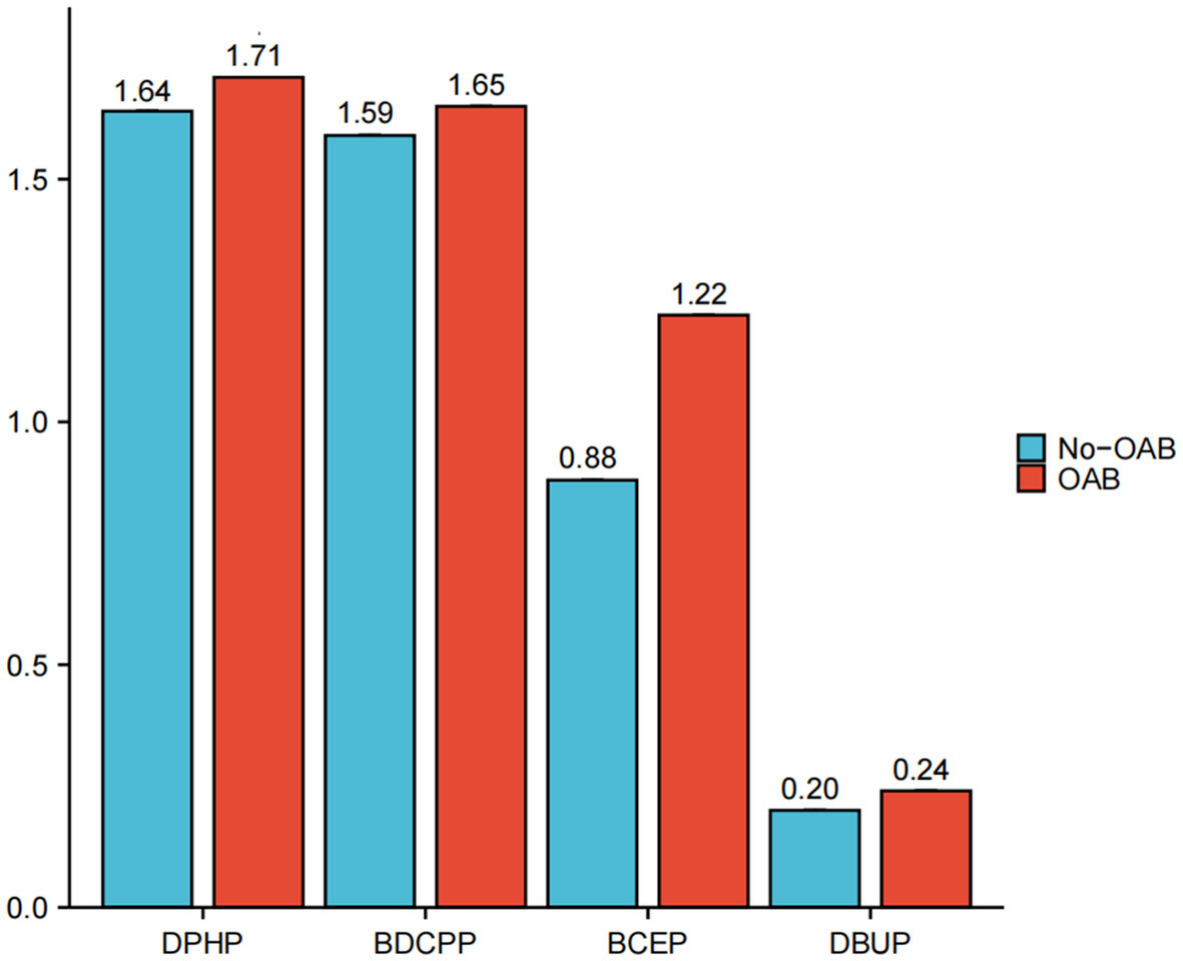
Urinary creatinine-corrected OPE metabolites exposure levels among 3,433 participants. Diphenyl phosphate (DPHP), Bis(1,3-dichloro-2-propyl) phosphate (BDCPP), Bis(2-chloroethyl) phosphate (BCEP), Dibutyl phosphate (DBUP).

Figure 1 illustrates the exposure levels of four distinct types of urinary, creatinine-adjusted organophosphate esters (OPEs) across 3,433 participants. The findings reveal that participants diagnosed with OAB present higher average exposure levels to DPHP, BDCPP, BCEP, and DBUP compared to those participants without the condition. Notably, among participants with overactive bladder, DPHP exhibited a markedly high exposure level of 1.71 ug/g, while DBUP demonstrated the lowest exposure level of 0.24 ug/g.

As shown in Table 2, in this study, a total of 3,433 people, 1,715 males and 1,718 females, were included. The prevalence of OAB was 17.39%, and statistically significant differences were found between the two groups in the distributions of age, race, marital status, educational levels, BMI, PIR, smoking status, drinking status, recreational activity, stroke, hypertension, and diabetes. Participants with OAB status were older than those without OAB. In addition, women, white, people with a college degree or above, obesity, drinking, physical inactivity, and high blood pressure were more likely to have overactive bladder.

**Table 2:** Baseline characteristics of the study population.

**Characteristic**	**Total**	**Overactive bladder**		***P*-value**
		**No**	**Yes**	
	**No. (%)**	**No. (%)**	**No. (%)**	
Total patients	3,433	2,836 (82.61)	597 (17.39)	
Age, years	44.57 (0.39)	41.18 (0.34)	60.21 (0.53)	**< 0.0001**
**Sex**				0.17
Male	1,715 (49.89)	1,466 (50.68)	249 (44.63)	
Female	1,718 (50.11)	1,370 (49.32)	348 (55.37)	
**Race**				**< 0.0001**
White	1,345 (66.46)	1,160 (67.58)	185 (58.99)	
Black	745 (10.76)	543 (9.37)	202 (20.03)	
Mexican	462 (8.29)	378 (8.30)	84 (8.18)	
Other	881 (14.49)	755 (14.75)	126 (12.79)	
**Marital status**				**0.002**
Married/living with partner	2,020 (63.27)	1,729 (64.49)	291 (55.14)	
Single/divorced/widowed	1,413 (36.73)	1,107 (35.51)	306 (44.86)	
**Education level**				**< 0.0001**
Less than high school	693 (13.59)	489 (11.93)	204 (24.68)	
High school or equivalent	772 (21.07)	621 (20.63)	151 (24.02)	
College or above	1,968 (65.34)	1,726 (67.44)	242 (51.30)	
**BMI**				**< 0.0001**
< 25	997 (29.34)	888 (30.83)	109 (19.44)	
25–30	1,105 (32.13)	935 (32.54)	170 (29.37)	
≥ 30	1,331 (38.53)	1,013 (36.63)	318 (51.19)	
**PIR**				**< 0.0001**
≤ 1.30	1,160 (22.96)	878 (20.96)	282 (36.25)	
1.31–3.50	1,247 (34.25)	1,040 (34.18)	207 (34.74)	
>3.50	1,026 (42.79)	918 (44.86)	108 (29.01)	
**Smoking status**				**0.02**
No	1,939 (57.61)	1,646 (58.98)	293 (48.50)	
Former	763 (22.94)	601 (22.08)	162 (28.68)	
Now	731 (19.45)	589 (18.94)	142 (22.81)	
**Drinking status**				**< 0.0001**
No	503 (11.72)	400 (11.19)	103 (15.30)	
Former	548 (13.03)	391 (11.69)	157 (21.95)	
Now	2,382 (75.25)	2,045 (77.13)	337 (62.75)	
**Recreational activity**				**< 0.0001**
Inactive or moderate	2,562 (71.05)	2,032 (68.60)	530 (87.35)	
Vigorous	871 (28.95)	804 (31.40)	67 (12.65)	
**Stroke**				**< 0.001**
No	3,341 (97.52)	2,781 (98.06)	560 (93.97)	
Yes	92 (2.48)	55 (1.94)	37 (6.03)	
**Hypertensio**				**< 0.0001**
No	2,077 (65.38)	1,857 (68.06)	220 (47.53)	
Yes	1,356 (34.62)	979 (31.94)	377 (52.47)	
**Diabetes**				**< 0.0001**
No	2,855 (87.75)	2,448 (89.47)	407 (76.35)	
Yes	578 (12.25)	388 (10.53)	190 (23.65)	
Creatinine urine (mg/dl)	121.02 (2.41)	121.53 (2.49)	117.64 (5.38)	0.48

BMI, body mass index; PIR, poverty income ratio.

Table 3 displays the results of multivariate logistic regression analyses. The results showed a positive relationship between the exposure level of DPHP and the risk of OAB. This association is significant in our model 1 (OR = 1.11; 95% CI = 1.02–1.22, *P* = 0.02) and model 2 (OR = 1.18; 95% CI = 1.06–1.32, *P* = 0.004). Furthermore, in Model 3, after adjusting for all covariates, these increases remained statistically significant (OR = 1.19; 95% CI = 1.06–1.32, *P* = 0.004). At the same time, we convert four types of OPEs from a continuous variable to a categorical variable (quartiles) for further analysis. After adjusting for all confounding factors, we observed a 1.67-fold and 1.75-fold increase in the risk of OAB in the DPHP Q3 and Q4 groups compared with the DPHP Q1 group (Q3 vs. Q1, OR = 1.67, 95%CI: 1.10–2.53, *P* = 0.02; Q4 vs. Q1, OR = 1.75,95%CI: 1.26–2.43). However, no statistically significant associations were observed between BDCPP, BCEP, and DBUP and OAB. Meanwhile, after dividing the participants by gender, only the female group retained the consistent results (OR = 1.05; 95% CI, 0.90–1.23; *P* = 0.04 in model 1, OR = 1.16; 95% CI: 0.98–1.36; *P* = 0.03 in model 2 and OR = 1.16; 95% CI, 0.98–1.36; *P* = 0.01 in model 3) (Supplementary Table 1). In contrast, no statistically significant association was observed between DPHP and OAB in the individual male group (Supplementary Table 2).

**Table 3:** Association of OPE metabolites (ln-transformed) and overactive bladder.

	**Model 1**	***P*-value**	**Model 2**	***P*-value**	**Model 3**	***P*-value**
	OR (95%CI)		OR (95%CI)		OR (95%CI)	
DPHP	1.11 (1.02,1.22)	**0.02**	1.18 (1.06,1.32)	**0.004**	1.19 (1.06,1.32)	**0.004**
**Stratified by DPHP quartiles**						
Quartile1	1		1		1	
Quartile2	1.06 (0.76,1.47)	0.74	1.20 (0.82,1.74)	0.33	1.19 (0.82,1.73)	0.34
Quartile3	1.35 (0.94,1.95)	0.10	1.70 (1.12,2.58)	**0.01**	1.67 (1.10,2.53)	**0.02**
Quartile4	1.42 (1.06,1.90)	**0.02**	1.75 (1.26,2.42)	**0.001**	1.75 (1.26,2.43)	**0.002**
BDCPP	0.97 (0.86,1.09)	0.58	1.12 (0.98,1.28)	0.09	1.12 (0.98,1.29)	0.10
**Stratified by BDCPP quartiles**						
Quartile1	1		1		1	
Quartile2	0.99 (0.73,1.34)	0.96	1.31 (0.89,1.91)	0.16	1.30 (0.89,1.91)	0.17
Quartile3	0.92 (0.63,1.34)	0.66	1.23 (0.80,1.88)	0.34	1.21 (0.79,1.88)	0.37
Quartile4	0.98 (0.73,1.33)	0.90	1.59 (1.11,2.29)	0.07	1.60 (1.10,2.33)	0.06
BCEP	1.04 (0.96,1.12)	0.34	1.00 (0.91,1.09)	0.96	1.00 (0.92,1.10)	0.94
**Stratified by BCEP quartiles**						
Quartile1	1		1		1	
Quartile2	1.17 (0.84,1.62)	0.35	1.33 (0.90,1.98)	0.15	1.35 (0.90,2.05)	0.14
Quartile3	1.15 (0.78,1.68)	0.47	1.16 (0.71,1.90)	0.54	1.17 (0.72,1.90)	0.52
Quartile4	1.16 (0.88,1.53)	0.28	1.02 (0.74,1.42)	0.89	1.04 (0.74,1.46)	0.80
DBUP	0.94 (0.82,1.08)	0.37	0.84 (0.72,0.98)	0.13	0.83 (0.71,0.97)	0.15
**Stratified by DBUP quartiles**						
Quartile1	1		1		1	
Quartile2	0.89 (0.68,1.16)	0.37	0.80 (0.59,1.09)	0.14	0.79 (0.57,1.09)	0.14
Quartile3	0.82 (0.55,1.22)	0.32	0.72 (0.44,1.16)	0.15	0.71 (0.44,1.14)	0.15
Quartile4	0.86 (0.60,1.24)	0.41	0.64 (0.42,0.98)	0.08	0.62 (0.41,0.95)	0.06

Model 1: unadjusted.

Model 2: adjusted for age, sex, race, marital status, education level, PIR, and BMI.

Model 3: further adjusted for recreational avtivity, smoking status, drinking status, stroke, hypertension as well as diabetes.

**Figure 2:**
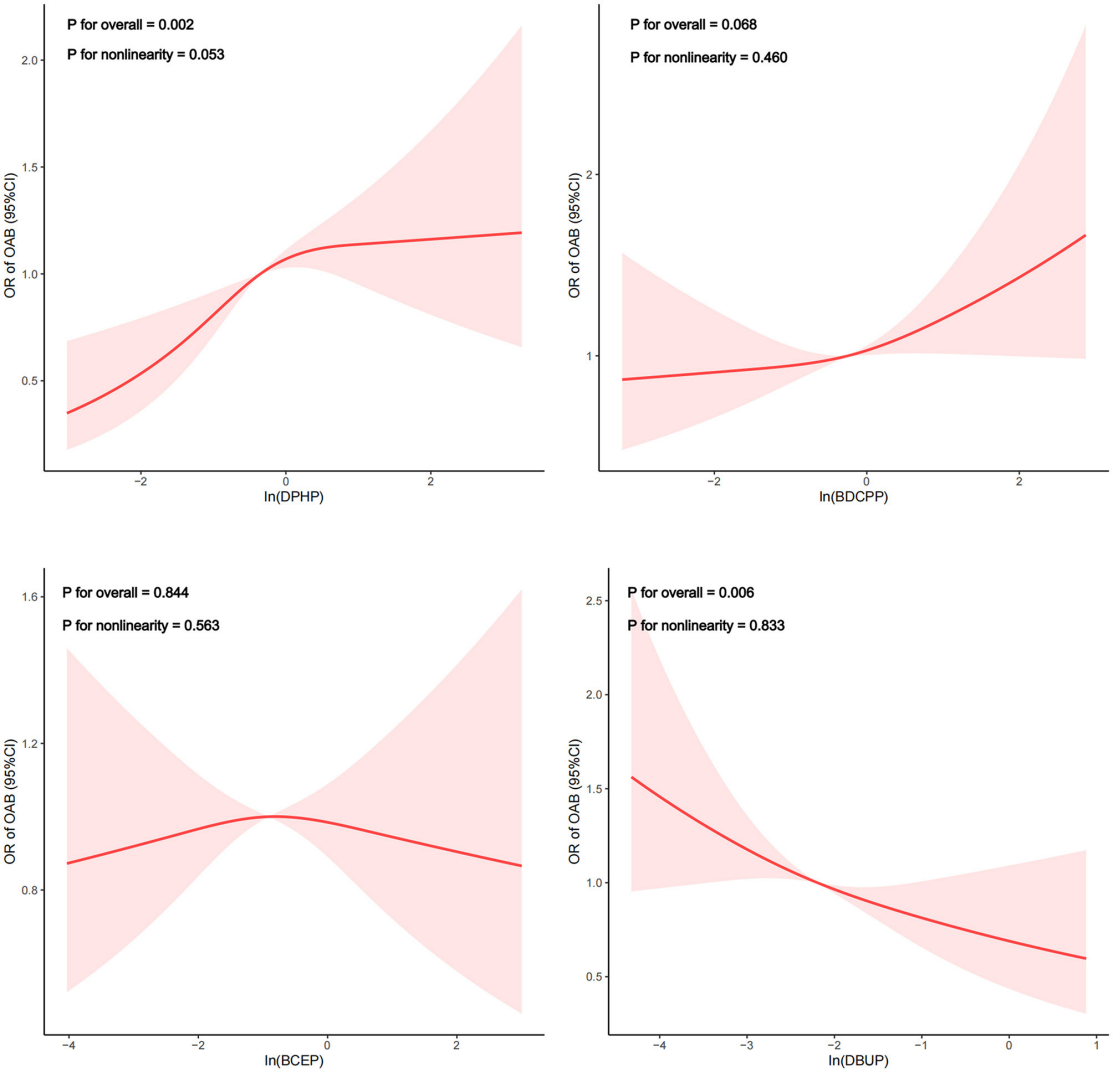
Dose-response relationship analysis between OPE metabolites and overactive bladder. Restricted cubic spline plots of the association between ln-transformed concentration of OPEs and OAB. RCS regression was adjusted for age, sex, race, marital status, educational levels, BMI, PIR, smoking status, drinking status, recreational activity, stroke, hypertension and diabetes (Model 3). The red or blue solid line represents ORs, red or blue shaded region represents 95 % CI.

The restricted cubic spline depicted the dose-response relationship analysis between the concentrations of DPHP and OAB in Figure 2. We detected that the risk of OAB gradually increased as DPHP increased (*P* for overall = 0.002; *P* for nonlinearity = 0.053). Meanwhile, a linear negative correlation between DBUP and OAB was observed (*P* for overall = 0.006; *P* for nonlinearity = 0.833). However, no linear or nonlinear associations were observed between BDCPP and BCEP and OAB. (Both *p* for overall > 0.05, both *p* for nonlinearity > 0.05). In addition, as shown in Supplementary Figure 2, a nonlinear correlation between DPHP and OAB was observed in females (*P* for overall = 0.002; *P* for nonlinearity = 0.002). Piecewise regression analysis demonstrated that the inflection point of In(DPHP) was 0.458 (DPH*P* = 1.581 ug/g) in female participants. Specifically, when the urinary creatinine adjusted DPHP was <1.581 ug/g, the risk of OAB increased with the level of exposure to DPHP. However, when urine creatinine adjusted DPHP is >1.581 ug/g, the risk of OAB is a downward trend. However, in male participants, no linear or non-linear association between DPHP and OAB was observed (*P* for overall > 0.05, *P* for nonlinearity > 0.05).

**Figure 3:**
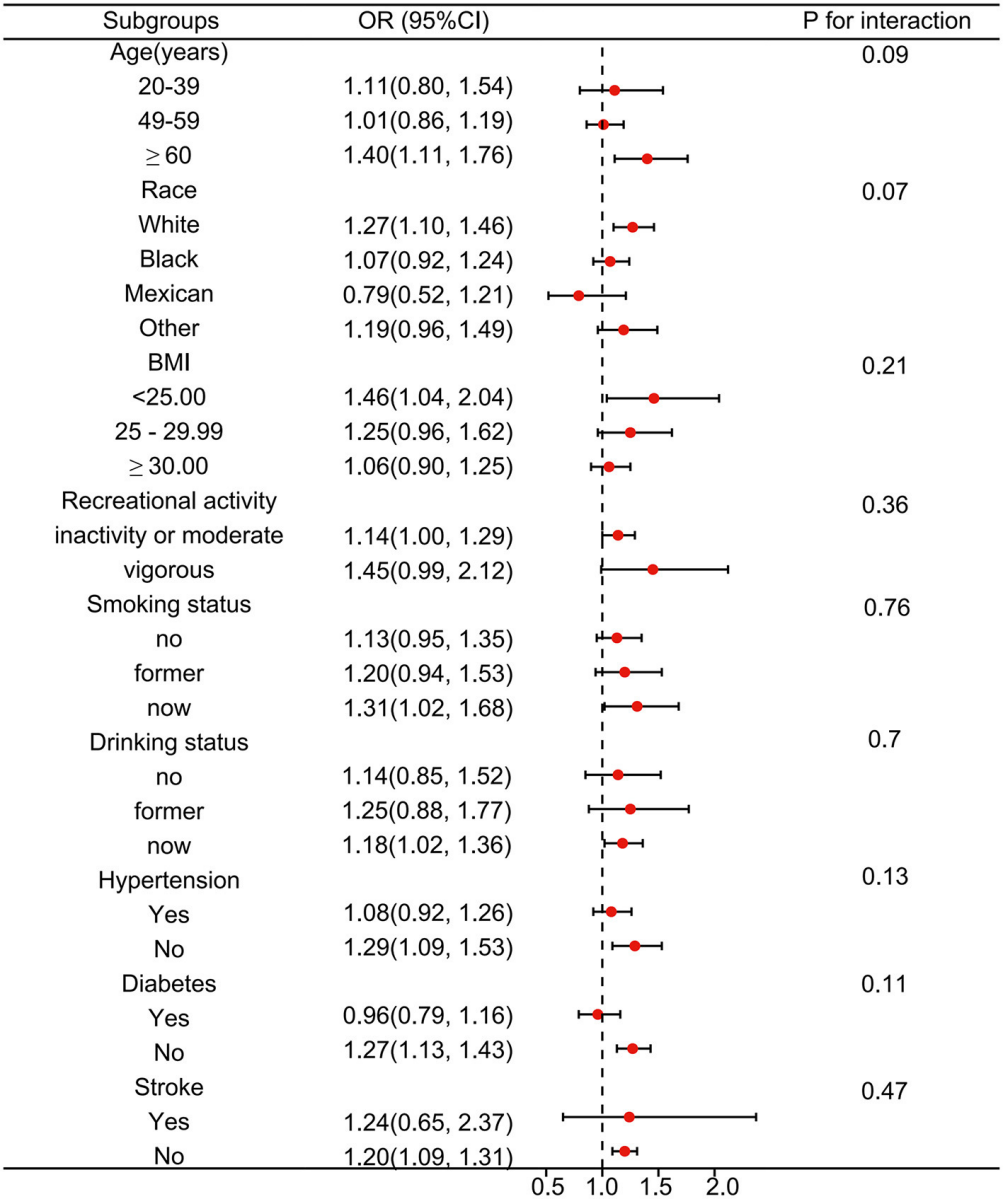
Subgroup analysis between Diphenyl phosphate (DPHP) and overactive bladder. Analyses were adjusted for age, sex, race, marital status, educational levels, BMI, PIR, smoking status, drinking status, recreational activity, stroke, hypertension and diabetes.

We further adopted stratified analysis to assess whether the correlation of DPHP with OAB was stable in different subgroups (Figure 3). After adjusting for the covariates, we found that there was no significant difference between OAB and DPHP within the subgroup. In detail, the associations between DPHP and the risk of OAB remained consistent in different subgroups by age, race, BMI, recreational activity, smoking status, drinking status, hypertension, diabetes, and stroke.

## 4. Discussion

Overactive bladder is a common urological disease, which not only brings a significant negative impact on a patient's quality of life but also leads to an economic loss of productivity and higher health care costs (31). Therefore, identifying the risk factors of OAB is of great significance for its prevention of it. To the best of our knowledge, our study is the first to examine the relationship between metabolites of OPEs and OAB. In this nationally representative study, we found a strong association between DPHP and OAB. Univariate and multivariate logistic regression results showed that exposure to high levels of DPHP was associated with a higher risk of OAB. When we converted DPHP from a continuous variable to a categorical variable, we found that higher DPHP exposure levels were significantly associated with higher OAB risk compared to lower DPHP exposure levels. However, when stratified by sex, this association remained consistent only among female participants. Dose-response analyses of DPHP and OAB indicated a nonlinear association between DPHP and OAB in female participants. Subgroup analysis by age, race, BMI, recreational activity, smoking status, drinking status, hypertension, diabetes, and stroke also showed no effect on the stability of the relationship between DPHP and OAB.

Research conducted over the past decade has demonstrated that OPEs have become almost ubiquitous in a range of microenvironments and populations worldwide. Exposure can arise from many common sources, including consumer products, construction materials, and food packaging (32). It is persistent in the environment and toxic to humans and animals. Numerous studies have demonstrated that OPEs may cause various side effects, including neurotoxicity, carcinogenesis, endocrine disrupting activity, reproductive, and developmental toxicity, among others (33–35). However, research examining the relationship between OPE exposure and bladder dysfunction in humans is limited. A recent analysis of the NHANES database by He et al. (15) found that urine metabolites of OPEs were linked to a higher odds ratio for mixed incontinence in females. Nonetheless, the precise mechanism by which OPEs impact bladder function remains unclear.

At present, it is increasingly evident that OPEs are a type of endocrine disruptor (36). It is hypothesized that OPEs may impact bladder function by interfering with endocrine pathways. Currently, there is scientific consensus that metabolic syndrome (MetS) and OAB may have shared pathophysiologies (37). A study conducted by Kai Luo et al. showed that exposure to OPEs elevated the risk of MetS in adults (14). At the same time, Yacong Bo and Kai Luo et al. discovered a positive correlation between organophosphate esters (OPEs) and insulin resistance in adults and adolescents (17, 38). Animal studies have also shown that OPE exposure can lead to insulin resistance (39). Uzun et al. (40) observed a link between insulin resistance and overactive bladder (OAB) in the population. Additionally, in an obese mice model, insulin resistance in the bladder mucosa impeded detrusor relaxation and contributed to bladder overactivity (41, 42). Therefore, OPEs might lead to overactive bladder by causing insulin resistance of the bladder mucosa.

Another possible mechanism is that OPEs may increase the risk of overactive bladder by affecting human sex hormone levels. Previous studies have demonstrated that male participants with higher DPHP urine levels had lower total testosterone and estradiol levels (43). Gao et al. (44) also found that some organophosphate esters such as TPhP and EHDPP may reduce estradiol levels in women of childbearing age. However, testosterone and estrogen can bind to corresponding receptors in the bladder and pelvic floor muscles to further affect bladder function (45, 46). The mechanism by which testosterone and estrogen affect bladder function was further elucidated in the mouse model. Kiril L and Georgi V Petkov discovered that testosterone and estradiol can lower the excitability of detrusor smooth muscle cells by directly activating BK channels through a nongenomic mechanism (47, 48). Other studies have demonstrated that reduced testosterone can cause fibrosis of the bladder wall in rats and impact the release of urinary mediators in the bladder, resulting in bladder dysfunction (49, 50). Additionally, animal studies have illustrated that estradiol may reverse urothelial damage, inflammatory cell infiltration, and muscular atrophy (51). Therefore, prolonged exposure to OPEs could result in reduced levels of estrogen and testosterone in the body, ultimately causing bladder dysfunction.

This study has several strengths and limitations. The main advantage is that the study includes a large population sample, which can be representative of the characteristics of the national population. In addition, we considered appropriate sampling weights in the analysis to reduce the bias of oversampling, which made our conclusions more reliable. This study also has some limitations. First, This is a cross-sectional survey, and the causal relationship between OPEs and the occurrence of OAB cannot be determined. Second, The information provided by NHANES on overactive bladder is incomplete and the diagnosis of OAB is mainly based on questionnaire form, which reduces accuracy. Finally, we cannot determine whether there are potential confounding factors that were not involved in the study.

## 5. Conclusions

Our results suggest that exposure to high levels of DPHP may increase the risk of OAB in US adult females. Further experimental studies are needed to explore its underlying mechanism in the future.

## Data availability statement

The datasets presented in this study can be found in online repositories. The names of the repository/repositories and accession number(s) can be found below: https://www.cdc.gov/nchs/nhanes/index.htm.

## Author contributions

WL and M-EL wrote, revised, and reviewed the manuscript, drafted the study design, and supervised all processes. HW and ZW performed the statistical analysis and interpreted the analysis. WZ wrote, revised, and reviewed the manuscript. All authors have reviewed the results and approved the submission of the manuscript.
