# Supplementary material for: Associations between exposure to organophosphate esters and overactive bladder in U.S. adults: a cross-sectional study
Source: Front Public Health. 2023 Nov 9;11:1186848. doi: 10.3389/fpubh.2023.1186848 (PMC10666646; doi:10.3389/fpubh.2023.1186848)
Supplement: Supplementary file 1 [file Data_Sheet_1.docx]

**Supplementary Figure 1 Directed acyclic graph of the associations between OPEs and overactive bladder**

**
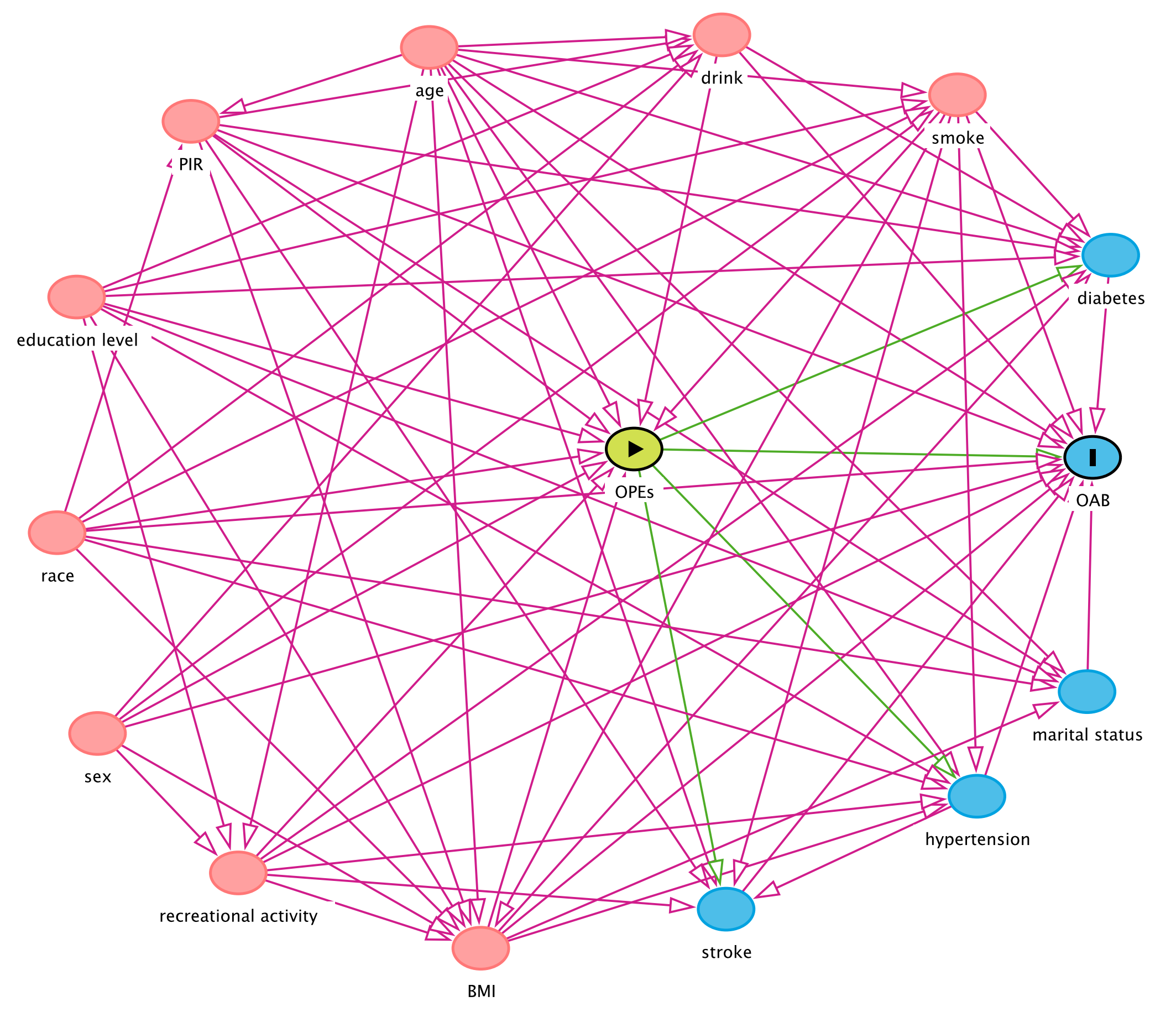
**

**Supplementary Table 1 Association of OPE metabolites (ln-transformed) and overactive bladder in female**

|  | Model 1 | P-value | Model 2 | P-value | Model 3 | P-value |
| --- | --- | --- | --- | --- | --- | --- |
|  | OR (95%CI) |  | OR (95%CI) |  | OR (95%CI) |  |
| DPHP | 1.05(0.90,1.23) | **0.04** | 1.16(0.98,1.36) | **0.03** | 1.16(0.98,1.36) | **0.01** |
| Stratified by DPHP quartiles | | | | | | |
| Quartile1 | 1 |  | 1 |  | 1 |  |
| Quartile2 | 1.22(0.78,1.91) | 0.37 | 1.53(0.94,2.48) | 0.33 | 1.48(0.90,2.42) | 0.34 |
| Quartile3 | 1.74(1.13,2.67) | 0.11 | 2.59(1.51,4.44) | **0.01** | 2.52(1.48,4.29) | **0.01** |
| Quartile4 | 1.32(0.84,2.06) | **0.01** | 1.92(1.18,3.14) | **0.001** | 1.92(1.17,3.15) | **0.001** |
|  | | | | | | |
| BDCPP | 1.00(0.87,1.15) | 0.99 | 1.16(0.97,1.37) | 0.10 | 1.17(0.99,1.40) | 0.07 |
| Stratified by BDCPP quartiles | | | | | | |
| Quartile1 | 1 |  | 1 |  | 1 |  |
| Quartile2 | 1.08(0.67,1.73) | 0.75 | 1.35(0.76,2.42) | 0.30 | 1.33(0.74,2.41) | 0.33 |
| Quartile3 | 0.92(0.61,1.41) | 0.71 | 1.10(0.65,1.85) | 0.71 | 1.08(0.63,1.86) | 0.76 |
| Quartile4 | 1.13(0.71,1.80) | 0.59 | 1.70(0.97,3.00) | 0.06 | 1.75(0.99,3.08) | 0.05 |
|  | | | | | | |
| BCEP | 0.99(0.89,1.09) | 0.80 | 0.94(0.85,1.05) | 0.28 | 0.94(0.85,1.05) | 0.26 |
| Stratified by BCEP quartiles | | | | | | |
| Quartile1 | 1 |  | 1 |  | 1 |  |
| Quartile2 | 1.27(0.71,2.27) | 0.41 | 1.45(0.73,2.87) | 0.28 | 1.52(0.79,2.94) | 0.20 |
| Quartile3 | 0.96(0.59,1.56) | 0.87 | 0.91(0.51,1.63) | 0.74 | 0.93(0.53,1.62) | 0.78 |
| Quartile4 | 1.00(0.67,1.50) | 0.99 | 0.87(0.56,1.34) | 0.51 | 0.86(0.58,1.30) | 0.47 |
|  | | | | | | |
| DBUP | 0.82(0.70,0.97) | 0.07 | 0.83(0.70,0.98) | 0.06 | 0.82(0.69,0.98) | 0.06 |
| Stratified by DBUP quartiles | | | | | | |
| Quartile1 | 1 |  | 1 |  | 1 |  |
| Quartile2 | 0.82(0.51,1.31) | 0.40 | 0.86(0.53,1.39) | 0.52 | 0.85(0.53,1.37) | 0.50 |
| Quartile3 | 0.64(0.43,0.95) | 0.12 | 0.70(0.44,1.11) | 0.12 | 0.69(0.43,1.09) | 0.11 |
| Quartile4 | 0.65(0.41,1.02) | 0.06 | 0.65(0.40,1.06) | 0.08 | 0.64(0.38,1.06) | 0.08 |

Model1: unadjusted

Model2: adjusted for age, race, marital status, education level, PIR, and BMI

Model3: further adjusted for recreational avtivity, smoking status, drinking status, stroke, hypertension as well as diabetes.

**Supplementary Table 2 Association of OPE metabolites (ln-transformed) and overactive bladder in male**

|  | Model 1 | P-value | Model 2 | P-value | Model 3 | P-value |
| --- | --- | --- | --- | --- | --- | --- |
|  | OR (95%CI) |  | OR (95%CI) |  | OR (95%CI) |  |
| DPHP | 1.15(0.91,1.44) | 0.24 | 1.19(0.92,1.54) | 0.17 | 1.19(0.91,1.55) | 0.20 |
| Stratified by DPHP quartiles | | | | | | |
| Quartile1 | 1 |  | 1 |  | 1 |  |
| Quartile2 | 0.94(0.55,1.61) | 0.81 | 0.95(0.52,1.71) | 0.85 | 0.95(0.54,1.69) | 0.87 |
| Quartile3 | 0.95(0.61,1.49) | 0.82 | 0.99(0.57,1.73) | 0.97 | 0.97(0.56,1.70) | 0.92 |
| Quartile4 | 1.64(0.86,3.12) | 0.13 | 1.82(0.86,3.88) | 0.12 | 1.80(0.83,3.93) | 0.13 |
|  | | | | | | |
| BDCPP | 0.90(0.69,1.17) | 0.43 | 1.07(0.83,1.38) | 0.59 | 1.07(0.82,1.38) | 0.61 |
| Stratified by BDCPP quartiles | | | | | | |
| Quartile1 | 1 |  | 1 |  | 1 |  |
| Quartile2 | 0.91(0.54,1.54) | 0.72 | 1.20(0.65,2.22) | 0.56 | 1.22(0.66,2.23) | 0.52 |
| Quartile3 | 0.91(0.49,1.69) | 0.77 | 1.50(0.75,2.99) | 0.24 | 1.49(0.75,2.93) | 0.24 |
| Quartile4 | 0.76(0.39,1.47) | 0.40 | 1.30(0.64,2.64) | 0.46 | 1.29(0.63,2.66) | 0.48 |
|  | | | | | | |
| BCEP | 1.08(0.93,1.27) | 0.30 | 1.07(0.90,1.27) | 0.46 | 1.07(0.90,1.27) | 0.41 |
| Stratified by BCEP quartiles | | | | | | |
| Quartile1 | 1 |  | 1 |  | 1 |  |
| Quartile2 | 1.02(0.54,1.94) | 0.94 | 1.18(0.58,2.41) | 0.63 | 1.18(0.57,2.45) | 0.65 |
| Quartile3 | 1.36(0.83,2.21) | 0.21 | 1.55(0.87,2.76) | 0.13 | 1.57(0.88,2.80) | 0.12 |
| Quartile4 | 1.32(0.72,2.41) | 0.36 | 1.25(0.63,2.49) | 0.52 | 1.27(0.65,2.49) | 0.47 |
|  | | | | | | |
| DBUP | 1.04(0.84,1.27) | 0.74 | 0.83(0.63,1.10) | 0.19 | 0.83(0.63,1.09) | 0.17 |
| Stratified by DBUP quartiles | | | | | | |
| Quartile1 | 1 |  | 1 |  | 1 |  |
| Quartile2 | 0.80(0.46,1.38) | 0.41 | 0.67(0.34,1.31) | 0.23 | 0.67(0.34,1.30) | 0.23 |
| Quartile3 | 0.95(0.52,1.73) | 0.86 | 0.71(0.35,1.46) | 0.35 | 0.71(0.36,1.42) | 0.32 |
| Quartile4 | 1.04(0.53,2.06) | 0.90 | 0.58(0.25,1.31) | 0.18 | 0.57(0.25,1.29) | 0.17 |

Model1: unadjusted

Model2: adjusted for age, race, marital status, education level, PIR, and BMI

Model3: further adjusted for recreational avtivity, smoking status, drinking status, stroke, hypertension as well as diabetes.

**Supplementary Figure 2 Dose-response relationship analysis between OPE metabolites and overactive bladder in different sex groups**


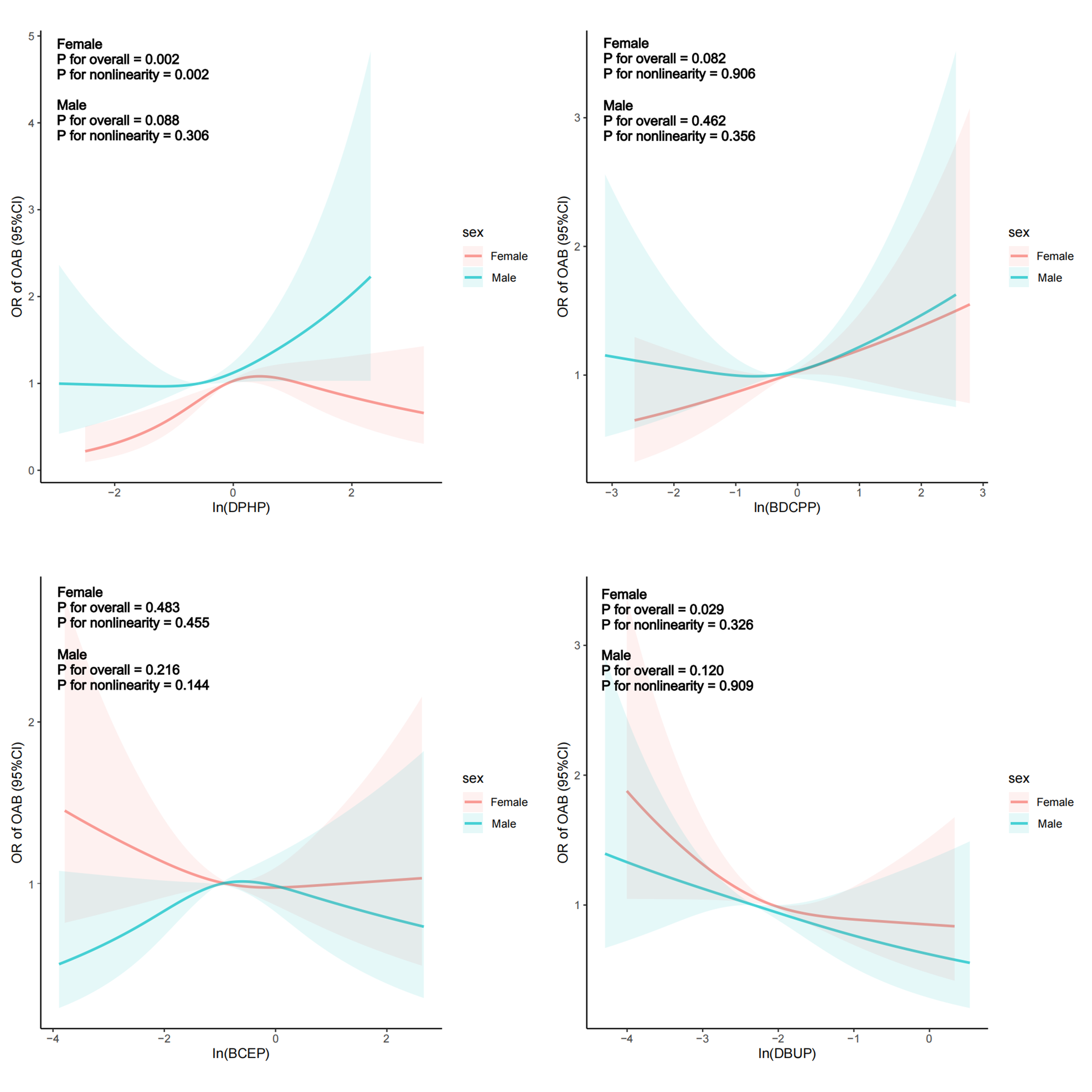


Restricted cubic spline plots of the association between ln-transformed concentration of DPHP and OAB. RCS regression was adjusted for age, sex, race, marital status, educational levels, BMI, PIR, smoking status, drinking status, recreational activity, stroke, hypertension and diabetes (Model 3).   The red or blue solid line represents ORs, red or blue shaded region represents 95 % CI.
